# Task-shifting HIV counselling and testing services in Zambia: the role of lay counsellors

**DOI:** 10.1186/1478-4491-7-44

**Published:** 2009-05-30

**Authors:** Parsa Sanjana, Kwasi Torpey, Alison Schwarzwalder, Caroline Simumba, Prisca Kasonde, Lameck Nyirenda, Paul Kapanda, Matilda Kakungu-Simpungwe, Mushota Kabaso, Catherine Thompson

**Affiliations:** 1 Family Health International/Zambia Prevention Care and Treatment Partnership, Lusaka, Zambia; 2 Columbia University Mailman School of Public Health, New York, New York, USA; 3 Ministry of Health/DHMT, Mansa, Zambia; 4 Ministry of Health/DHMT, Ndola, Zambia

## Abstract

**Background:**

The human resource shortage in Zambia is placing a heavy burden on the few health care workers available at health facilities. The Zambia Prevention, Care and Treatment Partnership began training and placing community volunteers as lay counsellors in order to complement the efforts of the health care workers in providing HIV counselling and testing services. These volunteers are trained using the standard national counselling and testing curriculum. This study was conducted to review the effectiveness of lay counsellors in addressing staff shortages and the provision of HIV counselling and testing services.

**Methods:**

Quantitative and qualitative data were collected by means of semistructured interviews from all active lay counsellors in each of the facilities and a facility manager or counselling supervisor overseeing counseling and testing services and clients. At each of the 10 selected facilities, all counselling and testing record books for the month of May 2007 were examined and any recordkeeping errors were tallied by cadre. Qualitative data were collected through focus group discussions with health care workers at each facility.

**Results:**

Lay counsellors provide counselling and testing services of quality and relieve the workload of overstretched health care workers. Facility managers recognize and appreciate the services provided by lay counsellors. Lay counsellors provide up to 70% of counselling and testing services at health facilities. The data review revealed lower error rates for lay counsellors, compared to health care workers, in completing the counselling and testing registers.

**Conclusion:**

Community volunteers, with approved training and ongoing supervision, can play a major role at health facilities to provide counselling and testing services of quality, and relieve the burden on already overstretched health care workers.

## Background

Zambia is among the countries hardest-hit by the HIV/AIDS epidemic in Africa. It is estimated that 1.2 million of the total Zambian population of 10 million was infected with HIV by 2005 [1,2]. Although declining HIV trends have been observed in young people since 1998, HIV/AIDS in Zambia is still a major threat to the lives of adults of reproductive age and their children [3]. Increasing access to HIV counselling and testing – the entry point to follow-on care, support and treatment services – could alter this trend.

Shortages of health care workers (HCWs) have been a bottleneck in the provision of HIV/AIDS services in resource-limited settings. The World Health Organization/Ministry of Health establishment recommends a staff population ratio of 1:5000, 1:700, and 1:8000 for doctors, nurses and pharmacists, respectively [4]. The existing human resource capacity in Zambia is far below the recommended cadre-to-population ratios, with existing levels of 1:17 589, 1: 8064 and 1:473 450 for doctors, nurses and pharmacists, respectively [4].

With the rapid expansion of access to antiretroviral therapy (ART), the increasing patient load will put a strain on the existing fragile human resource base. Universal access to ART treatment is inextricably linked to availability of and access to HIV counselling and testing (CT) services. However, the human resource shortage in Zambia, coupled with a national HIV prevalence rate of 14.3% [1], is placing a heavy burden on the few health care workers available at health facilities to provide these services.

In May 2005, Family Health International's Zambia Prevention, Care and Treatment Partnership (ZPCT), funded by the United States Agency for International Development (USAID) through the United States President's Emergency Plan for AIDS Relief (PEPFAR), began training and placing community volunteers as lay counsellors in order to complement the efforts of the HCWs in providing HIV counselling and help reduce their burden, using the national HIV CT curricula.

This national training package includes a two-week classroom component followed by a four-week supervised practicum component. The lay counsellors are certified after the practicum. The intensive training is able to address individual learning needs related to education or experience level.

The two-week classroom component of the training includes instruction as well as role-plays and case studies to ensure that trainees understand the concepts and methods of HIV counselling and testing. In the four-week practicum, each trainee practices counselling and testing under the supervision of an experienced CT provider. This ensures that each trainee is able to practise and refine his or her counselling skills. Each counsellor is certified only after successfully completing both the classroom and the practicum components of the training.

Lay counsellors were initially trained to provide only pre- and post-test HIV counseling, because HIV testing could be done only by HCWs. In May 2006, after certification of an original cohort of lay counsellors, the Zambian National HIV VCT guidelines were changed to allow non-health care workers to conduct HIV testing by means of finger-prick methodology. As a result, ZPCT began training all new and previously certified lay counsellors in HIV testing in addition to counselling.

Lay counsellors are selected based on their ability to read and write in English, residing within the facility catchment area, and experience with the health facility for at least one year. These criteria were used for both urban and rural settings, but preference was given to those with some level of background training in HIV/AIDS.

Prior to the introduction of lay counsellors, CT services were provided primarily by nurses during their free time. Existing human resource challenges and personnel shortages in many health facilities do not adequately address the importance of accessible, good-quality CT services. Most health facilities did not have staff dedicated to providing CT services – meaning that clients seeking the service may not have a HCW available to provide the service.

By focusing specifically on the CT aspect of HIV services, lay counsellors are able to devote more time to each client than are HCWs. Certified lay counsellors are placed in health facilities to provide services two to three days per week. Although officially certified and integrated into the operation of their facilities, lay counsellors are expected to maintain their status as community-level volunteers. The lay counsellor position is not part of the current MOH establishment. Under the direction of an appointed facility manager, lay counsellors provide CT services on a part-time basis under the supervision of facility managers. ZPCT furnishes a stipend of 100 000 Zambian kwacha per month (approximately USD 25) to cover travel expenses for days worked at the facility.

The objective of this study was to assess the effectiveness of lay counsellors in addressing human resource shortages in the provision of HIV CT services in selected health facilities. We also aimed to identify the extent and quality of the services provided by lay counsellors in health facilities and to assess differences in quality of services, client counselling satisfaction and accuracy of data recording.

## Methods

The study was conducted in 10 health facilities in two provinces in Zambia. A multi-stage purposive sampling process of two of the five provinces in which ZPCT operates was selected. Luapula Province lies in the northern section of the country and represents a rural population base with an overall density of 15.3 people/km^2 ^[5]. The second, Copperbelt Province, contains many of Zambia's larger urban areas and represents a population base with an overall density of 50.5 people/km^2 ^[5]. These two provinces – one predominantly urban and one predominantly rural – were selected in order to encompass variability in HIV prevalence rates as well as potential differences in the implementation and acceptability of lay counsellor CT services.

Within these two provinces, all ZPCT-supported health facilities in which lay counsellors had been trained, placed and active for at least one year prior to study initiation were selected for evaluation (a total of 10 health facilities). Four of the selected health facilities were located in Luapula Province, and six were located in Copperbelt Province.

This final sample included facilities serving a range of population catchment sizes and was composed of rural health centres, urban clinics and secondary- and tertiary-level government hospitals. Health facility staff selected participating lay counsellors from among volunteers with existing ties to the facility for at least one year. A list of the facilities selected for the study appears in Table 1.

**Table 1:** List of health facilities included in the study sample in Luapula and Copperbelt provinces, Zambia

Luapula Province	Copperbelt Province
Mansa General Hospital	Ndola Central Hospital
Mansa Central Urban Clinic	Chipokota Mayamba Clinic
Chembe Rural Health Centre	Lubuto Health Centre
Senama Health Centre	Mushili Clinic
	Kawama Health Centre Ndeke Clinic

The study used both quantitative and qualitative methods. By means of semistructured interviews, data were collected from all active ZPCT-trained lay counsellors in each of the facilities, a facility manager or counselling supervisor overseeing CT services and CT clients. All interviews were conducted at the facilities during times convenient to those interviewed; generally in-between clients or meetings for counsellors or managers, and between or after service appointments for clients. All those interviewed were reminded that their responses would be kept confidential and that the interpretation of their responses would be unbiased.

CT recordkeeping was evaluated as a quality assurance measure. At each of the 10 selected facilities, all CT record books for the month of May 2007 (one month prior to study initiation) were examined and any recordkeeping errors were tallied. Standardized logs and instructions are provided to each of the facilities to promote monitoring and evaluation as well as to record the number of clients seeking services and their basic demographics. The standardization of these logs allowed for the generation of a list of mistakes to be considered recording errors. This list of predetermined errors was then used to tally all those found in each logbook used at these 10 facilities. A total of 1083 entries was reviewed with a data accuracy checklist. In addition, CT uptake data from October 2005 to September 2006 and October 2006 to September 2007 before and after the introduction of lay counsellors were reviewed to assess service statistics trends.

Qualitative data were collected through focus group discussions with health care workers at each facility.

Table 2 provides a summary of the type and number of respondents and the data collection method used with each group of respondents.

**Table 2:** Summary of respondents and sample size

Respondents	Data Collection	Sample Size	Comments
Lay counsellors	Semistructured interview	19	Maximum number available at each site (1–3 counsellors per site)

Health care workers	Focus group discussion	16	Eight focus groups (one per health centre)

Health macility managers	Semistructured interview	10	One per site

CT health facility clients	Exit interview	95	Convenience sample of (5–11 clients per site)

Data collected from semistructured interviews with lay counsellors, clients and facility managers were analysed both quantitatively and qualitatively. All quantitative data analyses were performed with SAS statistical software, version 9.1 (SAS Institute, Cary, North Carolina, United States of America). All qualitative, open-ended question responses were coded by hand to look for common themes and then analysed to draw conclusions. Data gathered from the quality assurance portion of the evaluation were used to calculate error rates at the level of the facility and of the province. Since these errors were also tallied according to the initials of the recording CT provider, they were also split by provider to assess differences between lay counsellors and health care workers.

## Results

Of the 19 lay counselors interviewed, six were based at health facilities in Luapula and 13 at Copperbelt health facilities. The average age of the lay counsellors was 44.8 years, ranging from 32 to 59 years. Eleven of the lay counsellors interviewed were male (57.9%) and eight were female (42.1%). More than half (57.9%) of lay counsellors provided services at the health facility prior to training and placement as a lay counsellor. Fifteen lay counsellors (78.9%) still assist with other services at their assigned health facility, including community health education and assisting with child health days.

### Job motivation

Lay counsellors were dedicated to their work and consider themselves professionals. They were confident in their counselling skills and found their work rewarding. When asked about their main motivation for being a counsellor, the most common response given was the ability to "help people" and serve their community, as well as allusions to how HIV/AIDS had touched them personally.

"Lots of people are dying without knowing their status. We are role models and we can impart information...knowledge is power."

### Services by lay counsellors

The data revealed that 70.5% of CT clients interviewed received CT from a lay counsellor rather than a health care worker at the study sites. With an average of 2.4 lay counsellors at each facility visited (ranging from one to four), lay counsellors were available almost all the time to provide CT services. The lay counsellors spent an average of 2.8 days (range 2 to 5) at their assigned health facility, providing CT services to an average of 5.6 clients (range 3 to 8) per day.

### Quality of services

The quality of counselling provided by lay counsellors was high, and comparable to the CT services provided by HCWs. Table 3 provides a comparison of results from clients served by a lay counsellor and a HCW, showing that there is no difference across a number of factors assessed (p-value > 0.05). In addition, data indicate that clients who received CT services from a lay counsellor waited an average of almost 15 minutes less than clients who received CT services from a HCW.

**Table 3:** Comparison of counseling provided by lay counsellors and HCWs from client exit interviews (n = 95)

**Question**	**% of clients responding "yes" who were counseled by**		
		
	Lay Counsellors	HCWs	p-value
Did staff/counsellor fully explain what to expect at the CT site?	98.5%	96.4%	p > 0.05

Did the counsellor make you comfortable talking to him/her?	97.0%	100.0%	p > 0.05

Did the counsellor display good skills in his/her counselling session?	98.5%	96.4%	p > 0.05

Were you given the necessary information you need about HIV/AIDS?	94.0%	100.0%	p > 0.05

Did the counsellor help you to identify ways of reducing your exposure to HIV?	94.0%	100.0%	p > 0.05

Overall, were the services you received at the CT centre satisfactory?	97.0%	100.0%	p > 0.05

Facility managers also rated the CT services provided by lay counsellors as average to excellent. None rated the services as below average.

### Addressing the HCW workload and human resource issues

According to health facility managers interviewed, lay counsellors have contributed significantly to reducing the workload of HCWs, even having a "tremendous" or "overwhelming" impact.

"They have given us a relief, coverage of CT services has gone up and we have been able to reach our targets."

All facility managers interviewed mentioned that the presence of lay counsellors has resulted in more clients' gaining access to CT services, while simultaneously decreasing the workload of health care workers. Lay counsellors were always or usually available to provide CT services. As indicated in Table 4, uptake of CT services increased by about 27.3% and 101.3% in Luapula and Copperbelt provinces, respectively, after the introduction of lay counsellors.

**Table 4:** Uptake of CT services before and after the introduction of lay counsellors

Province	Number of clients counselled, tested and who received results		Percent increase
		
	Oct 2005 to Sept 2006 (Before)	Nov 2006 to Oct 2007 (After)	
Copperbelt	5298	10 665	101.3%

Luapula	5414	6893	27.3%

**Grand total**	**10 713**	**17 558**	**63.9%**

### Data quality

The review of CT records revealed that data accuracy was generally high among both lay counsellors and HCWs. The error rate for lay counsellors was lower than the error rate of HCWs (6.44, compared to 16.81 per 1,000 fields p < 0.05).

### Sustainability

Health center managers expressed concern about retention of lay counsellors:

"The drawback is the amount of money they receive. They are here for 2–3 days, all day, and with no lunch. What they receive is too little. We may lose them if they find better payment in the future. If they leave us, this will impact negatively."

In addition, sustaining the quality of services requires refresher training to maintain skills and knowledge. Although the training received by the lay counsellors was rated as "good" or "very good", additional training needs were identified by almost 85% of the lay counsellors interviewed.

## Discussion

This paper has presented results from a formative evaluation based on data record reviews as well as interviews with several groups of key programme stakeholders. This evaluation design was intended to capture the experience of those individuals who had been directly involved with programme implementation and who had used the lay counsellors' services.

The results support the conclusion that lay counsellors are actively providing services at ZPCT-supported facilities. We found a self-reported mean of 2.8 days spent at the facility each week, with some lay counsellors reporting that they spent as many as five days per week at their facility. We also estimated that lay counsellors are providing a significant proportion (average of 70.5%) of the CT services conducted at these facilities, based on data gathered from interviews with facility managers as well as tabulations from CT record books.

A major reason for the use of lay counsellors is the potential they have for relieving already-overburdened health care workers and increasing CT uptake rates. Medically trained nurses and physicians have numerous clinical responsibilities and often do not have the time to provide CT services, which can be time-consuming. Since lay counsellors are trained specifically and uniquely in CT, this degree of specialization allows them to focus exclusively on service provision of consistent quality, while allowing health care workers to concentrate on other aspects of clinical care.

Community volunteers, with approved training and ongoing supervision, can play a major role at health facilities to provide good-quality CT services and relieve the burden on already-overstretched HCWs. From the interviews of facility managers and clients, we found that the same facility managers endorsed the quality of the lay counsellors' work and that there was no difference in satisfaction level between CT clients counselled by lay counsellors as those counselled by other health care workers. These results support previous studies, which have shown lay counsellors to be acceptable CT providers and readily used by clients [6,7].

Additionally, our quality assurance assessment found that error rates in CT recordkeeping were lower for lay counsellors than for other health care workers. The combination of this set of findings indicates that lay counsellors are positively affecting the provision of CT services without compromising service quality or monitoring and evaluation standards.

A third important theme that emerged from these results arose from interviews conducted with lay counsellors themselves. During interviews, some lay counsellors spoke of their influence in lessening stigma as well as representing community role models. These understandings reinforce their position of importance within the community and add significant weight and responsibility to their specified duties. This interpretation also supports the findings of Grinstead and colleagues [8], which highlight the perception of similar obligations and responsibilities.

The broad conception of the lay counsellor role as situated within larger professional structures also appeared during these interviews. Almost all the lay counsellors we interviewed were interested in future training and continuing in what was considered a professional field, including obtaining advanced certificates and degrees. The lay counsellors have a deep sense of commitment to their role in the health facility and do view themselves as professionals providing a critical service, as evidenced by the in-depth interviews.

A final point of interest is the challenge of maintaining the long-term sustainability of the lay counsellor programme, as it has been implemented. As health facilities become increasingly dependent on community volunteers, the issue of sustainability must be critically examined. Interviews conducted with both facility managers and lay counsellors raised several programmatic issues that echo those found in the community health worker literature.

The first is the degree to which counsellor remuneration will continue to affect the course of this programme in terms of retention rates and the ultimate impact of lay counsellors on these facilities. Several facility managers and almost all lay counsellors felt that for a volunteer-based programme, a travel stipend was not enough compensation for the services provided. Maintaining a volunteer-based programme may force participants to choose between continuing as a lay counsellor and the economic necessity of finding additional paid employment elsewhere, a concern raised by Zachariah et al. [9].

Although participants were not asked directly about the issue of financial stability, many raised the conditional nature of their continuation in this programme. We can further speculate, although additional research would be needed, that more formalized job and payment structures are desired, given the extent of the training required for the provision of high-quality, HIV-specific services and in the context of strong beliefs regarding the important contributions that lay counsellors are making at the community level. These factors may serve to increasingly foster a professional identity around lay counselling.

## Conclusion

Lay counsellors, when provided with the approved and appropriate training, can play a key role in HIV counselling services. While they can support the provision of good-quality counselling and testing services to relieve overburdened health care workers, they will require ongoing supervision to further enhance their performance. In order to make this strategy sustainable, efforts must be made to mainstream their activities and formalize their relationship with the health facilities.

## Competing interests

The authors declare that they have no competing interests.

## Authors' contributions

PS, KT, AS and CS conceived the study, participated in the design and helped draft the manuscript. PK, LN, DK, MS, MK and CT participated in the design and helped draft the manuscript. LN and MK did the statistical analysis. All authors read and approved the final manuscript.
